# A network-based method for identifying prognostic gene modules in lung squamous carcinoma

**DOI:** 10.18632/oncotarget.7632

**Published:** 2016-02-23

**Authors:** Lin Feng, Run Tong, Xiaohong Liu, Kaitai Zhang, Guiqi Wang, Lei Zhang, Ning An, Shujun Cheng

**Affiliations:** ^1^ State Key Laboratory of Molecular Oncology, Department of Etiology and Carcinogenesis, Peking Union Medical College and Cancer Institute (Hospital), Chinese Academy of Medical Sciences, Beijing, China; ^2^ Department of Respiratory and Critical Care Medicine, China-Japan Friendship Hospital, Beijing, China; ^3^ Department of Gynecology and Obstetrics, Maternal and Child Health Care Hospital of Haidian, Beijing, China; ^4^ Department of Endoscopy, Cancer Hospital, Chinese Academy of Medical Sciences, Beijing, China

**Keywords:** lung squamous carcinoma, multi-stage carcinogenesis, network-based, greedy searching, prognostic module

## Abstract

Similarities in gene expression between both developing embryonic and precancerous tissues and cancer tissues may help identify much-needed biomarkers and therapeutic targets in lung squamous carcinoma. In this study, human lung samples representing ten successive time points, from embryonic development to carcinogenesis, were used to construct global gene expression profiles. Differentially expressed genes with similar expression in precancerous and cancer samples were identified. Using a network-based greedy searching algorithm to analyze the training cohort (*n* = 69) and three independent testing cohorts, we successfully identified a significant 22-gene module in which expression levels were correlated with overall survival in lung squamous carcinoma patients.

## INTRODUCTION

The initiation of lung squamous carcinoma (LSQC) is characterized by five major successive stages: normal bronchial epithelium, squamous metaplasia, mild-moderate dysplasia, severe dysplasia (carcinoma in situ), and invasive carcinoma [1]. Many genetic or epigenetic changes essential for cancer initiation have already taken place in precancerous bronchial lesions before cancer formation [2]. Cisplatin plus gemcitabine is still the first-line treatment for LSQC [3, 4], and therapeutic options for LSQC patients remain limited because no specific molecular targets have been identified [5]. Therefore, understanding the molecular alterations that occur during carcinogenesis, especially in precancerous stages, might aid in the discovery of prognostic biomarkers and identification of candidate therapeutic targets.

The association between embryonic development and carcinogenesis has been widely documented, and some molecules are essential in both processes. *Ptch1* is a key regulator of embryonic development, and its overexpression promotes skin carcinogenesis [6]. *Scrib*, a mediator of epidermal permeability barrier acquisition and skeletal morphogenesis during embryonic development, is a potent tumor suppressor in cutaneous carcinogenesis [7]. Developmental animal models have also been used to uncover complicated molecular mechanisms of carcinogenesis [8, 9]. For instance, the *Notch1* signaling pathway, which is activated during development, is reactivated during carcinogenesis [10, 11]. In addition, cancer gene expression profiles can recapitulate the expression patterns of embryonic development [12-17]. These findings suggest that tumors can be viewed as an aberrant organs which have acquired the capacity for indefinite proliferation through various genetic alterations [18].

In this study, expression profiles of human lung tissues at various stages from embryonic development to carcinogenesis were used to identify differentially expressed genes (DEGs) of interest. A prior knowledge-based biological network was used to identify gene module(s) correlated with overall survival (OS) in LSQC patients. Using a greedy searching algorithm, we successfully identified a 22-gene module for which expression was significantly correlated with OS.

## RESULTS

**Figure 1:**
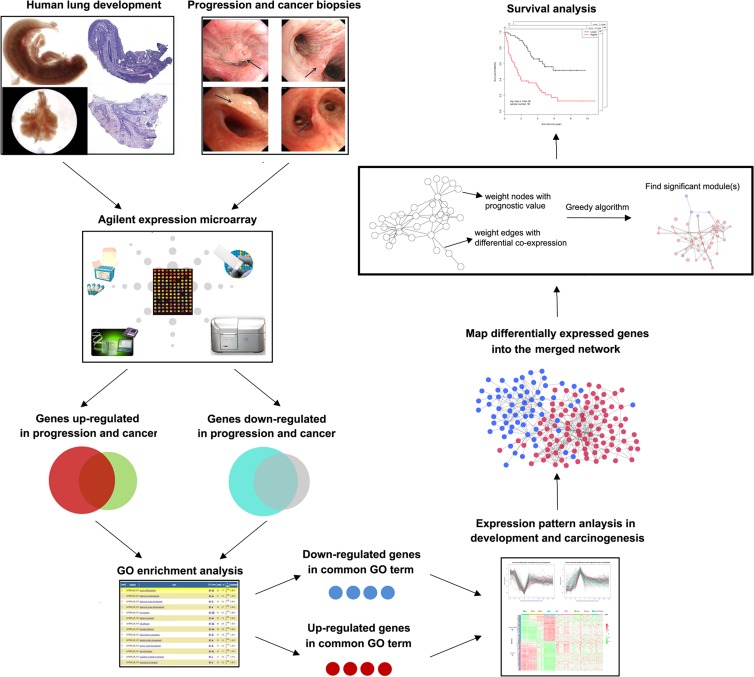
Schematic of methodology applied in this study Step I: Construction of global expression profiles of human lung embryonic development and LSQC carcinogenesis samples; Step II: Identification of consistent DEGs in precancerous and cancer samples; Step III: Using a greedy searching algorithm to identify module(s) significantly associated with overall survival.

A schematic for the study is depicted in Figure 1.

### Identification of DEGs consistently differentiated in both precancerous and cancer samples reduced signal noise

**Figure 2:**
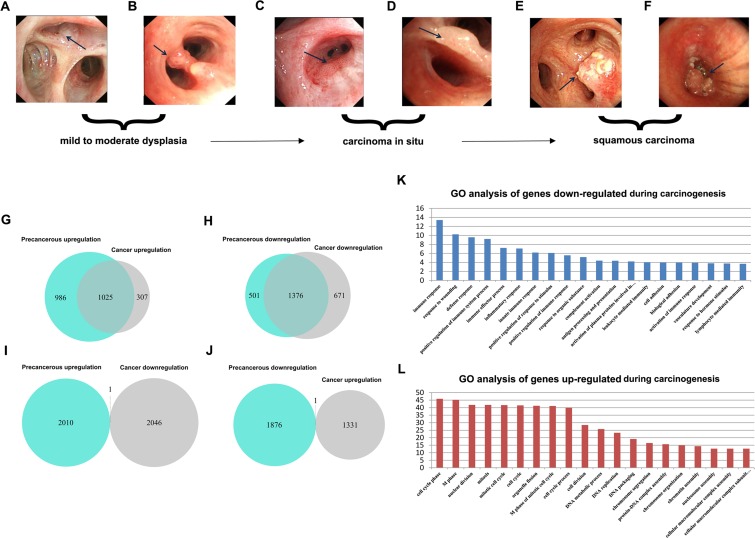
Identification of consistent DEGs and GO enrichment analysis **A.**-**F.** Bronchoscopic manifestation of multi-stage tissues during LSQC carcinogenesis. **G.**-**J.** Venn diagrams of the DEGs in precancerous and cancer stage samples. The majority of the genes differentiated in cancer were already consistently differentiated in the precancerous stage. G. Identification of 1025 consistent up-regulated DEGs. **H.** Identification of 1376 consistent down-regulated DEGs. **I.**-**J.** Only two DEGs were inconsistently differentiated in precancerous and cancer stages, probably as a result of noise. **K.** GO enrichment analysis of down-regulated consistent DEGs indicated these genes were associated with immune response. **L.** GO enrichment analysis of up-regulated consistent DEGs indicated that these genes were related to the cell cycle.

First, we analyzed the global expression profiles of human adult normal lung (NL), LSQC precancerous progression (Figure 2A-2D), and cancer samples (Figure 2E-2F) to identify DEGs of interest during carcinogenesis. 2011 genes were up-regulated and 1877 genes were down-regulated in precancerous samples in comparison to NL, and 1332 genes were up-regulated and 2047 genes were down-regulated in cancer samples compared to NL. Notably, a large portion of the DEGs differentiated in cancer were already consistently differentiated in precancerous stages (Figure 2G-2J). To reduce signal noise, DEGs that were up-regulated or down-regulated in both progression and cancer samples, referred to as consistent DEGs, were isolated. 1025 up-regulated (Figure 2G) and 1376 down-regulated (Figure 2H) consistent DEGs were identified.

### Consistent DEGs have roles in immune response and cell cycle processes

GO enrichment analysis was conducted *via* the DAVID bioinformatics tool (http://david.abcc.ncifcrf.gov/). The consistently down-regulated DEGs were functionally related to “immune response”, since the majority of the enriched GO terms for these genes were offspring of that GO term (*FDR* < 0.001, Figure 2K, Supplementary Table 1), while consistently up-regulated DEGs were functionally related to “cell cycle” (*FDR* < 0.001, Figure 2L, Supplementary Table 2). Therefore, we used 208 consistently down-regulated DEGs belonging to the GO term “immune response” (hereafter termed as “Immune DOWN” genes) and 234 consistently up-regulated DEGs belonging to the GO term “cell cycle” (hereafter termed as “Cycle UP” genes) for further analyses.

### Immune DOWN and Cycle UP genes were differentially regulated similarly in both embryonic development and carcinogenesis

**Figure 3:**
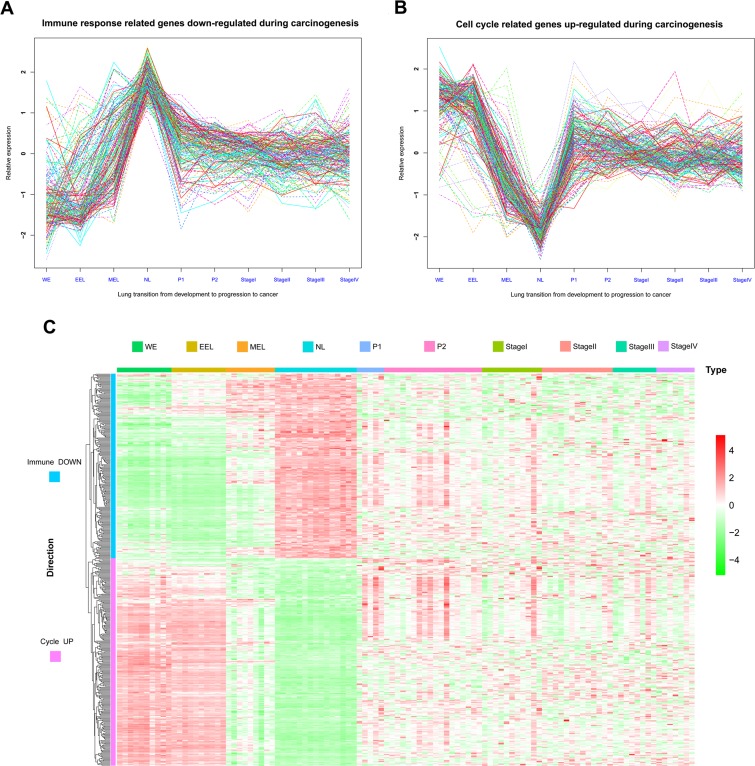
Matplots and heatmap of Immune DOWN and Cycle UP genes from embryonic development to cancer **A.** Matplot of Immune DOWN genes across ten time points showing that these genes were downregulated in both embryonic development and cancer. **B.** Matplot of Cycle UP genes, which were upregulated in both embryonic development and cancer. **C.** Heatmap of Immune DOWN and Cycle UP genes across ten time points. Rows represent genes, and columns represent samples from the ten time points. Genes were clustered with an unsupervised clustering algorithm (UCA). The two groups of genes were divided into two distinct clusters.

**Figure 4:**
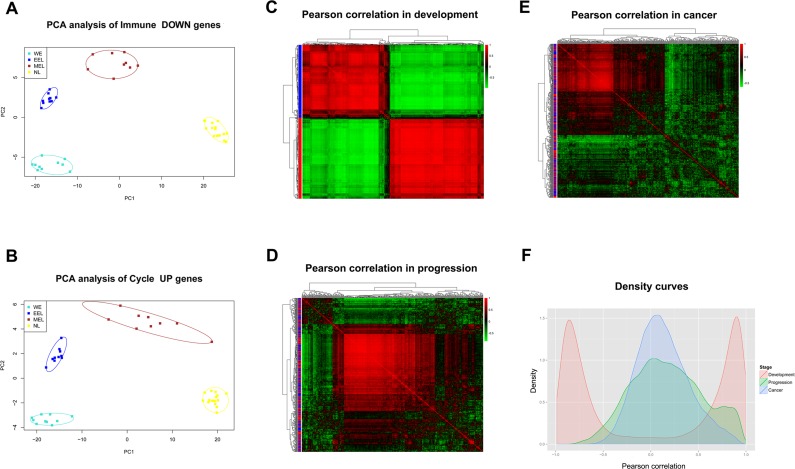
Gene expression and Pearson correlation pattern analyses **A.**-**B.** PCA analyses with Immune DOWN and Cycle UP genes recapitulating the trajectory of human lung development. The lung developmental samples clustered tightly within each developmental stage, but not between different stages, implying that the differentiation of these genes might promote human lung development. **C.**-**E.** Heatmaps of Pearson correlation patterns for Immune DOWN and Cycle UP genes in C. the development stage, D. the precancerous progression stage and E. the cancer stage. Red rows represent Cycle UP genes, while blue rows represent Immune DOWN genes. The heatmaps indicated that the co-expression pattern was continuously disrupted from development to carcinogenesis. F. Density curves of Pearson correlation in three stages. A bimodal distribution was seen for the developmental stage, in contrast to the unimodal distributions of the progression and cancer stages. Furthermore, the cancer stage had a higher maximum density at a Pearson correlation of zero compared with the progression or developmental stages, also indicating deteriorating gene-to-gene co-expression.

Expression profiles from human lung tissue during embryonic development [whole embryo (WE) at postovulatory weeks (PWs) 3 to 5, early embryonic lung (EEL) at 6 to 8 PWs, middle embryonic lung (MEL) at 16 to 24 PWs, and NL], LSQC precancerous progression [mild or moderate dysplasia (termed as P1) and carcinoma in situ (termed as P2)], and cancer (Stage I-IV) samples, were used to construct matplots of Immune DOWN (Figure 3A) and Cycle UP (Figure 3B) genes showing expression trajectories from embryonic development to carcinogenesis. A heatmap was also generated showing both Immune DOWN and Cycle UP genes across samples from all stages (Figure 3C). Immune genes down-regulated during carcinogenesis had the propensity to be kept increasing along embryonic development (underexpressed in developmental samples comparing to NL), while cell cycle genes up-regulated during carcinogenesis tended to be kept decreasing along development time-axis (overexpressed in developmental samples comparing to NL); and these two gene groups were divided into two distinct clusters (Figure 3). In addition, principle component analysis (PCA) of the development data indicated that human lung ontogenesis was characterized by sequential changes in transcriptomic features, and developmental trajectory was recapitulated by Immune DOWN (Figure 4A) and Cycle UP (Figure 4B) genes. Samples clustered tightly within each developmental stage, but differed between different stages (Figure 4A-4B). Moreover, according to gene set enrichment analysis (GSEA) conducted in 52 paired Cancer Genome Atlas (TCGA) samples (cancer and adjacent normal tissue), Immune DOWN genes were significantly down-regulated (Supplementary Figure 1A) and Cycle UP genes were significantly up-regulated (Supplementary Figure 1B) in cancer, which was highly consistent with our microarray results (comparing normal adult lung with cancer samples).

### Continuous co-expression disruption affected Immune DOWN and Cycle UP genes during carcinogenesis

Pairwise Pearson correlations among the Immune DOWN and Cycle UP genes (442 consistent DEGs in total) were calculated to construct diagonally symmetric Pearson correlation heatmaps for lung samples during embryonic development (Figure 4C), precancerous progression (Figure 4D), and cancer (Figure 4E) stages. During the developmental stage, Immune DOWN and Cycle UP genes were divided into two distinct clusters that were not present in the precancerous and cancer stages. Superimposing the three Pearson correlation density curves (Figure 4F) revealed a clear bimodal distribution for the developmental stage and unimodal distributions for the progression and cancer stages, suggesting that co-expression differentiation plays an important role during carcinogenesis.

### A network-based method identified a 22-gene module correlated with overall survival

**Figure 5:**
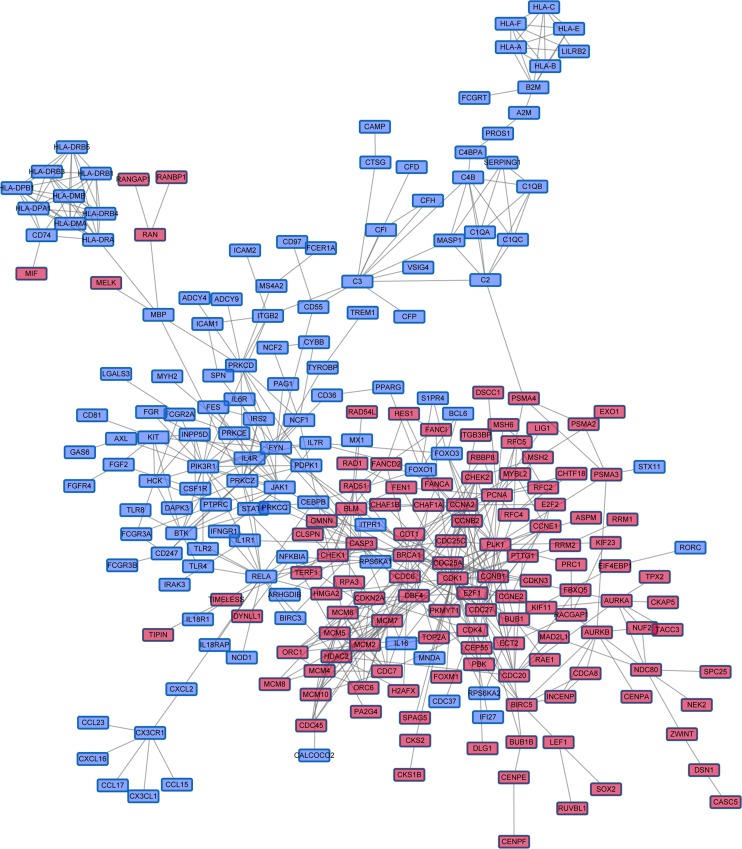
The largest Immune DOWN and Cycle UP gene component within the merged biological network Immune DOWN and Cycle UP genes were mapped into the merged biological network, and the largest connected component, containing 246 genes and 540 interactions, was analyzed further. These genes served as the initial gene pool for further module identification. Red nodes represent Cycle UP genes, while blue nodes represent Immune DOWN genes.

**Figure 6:**
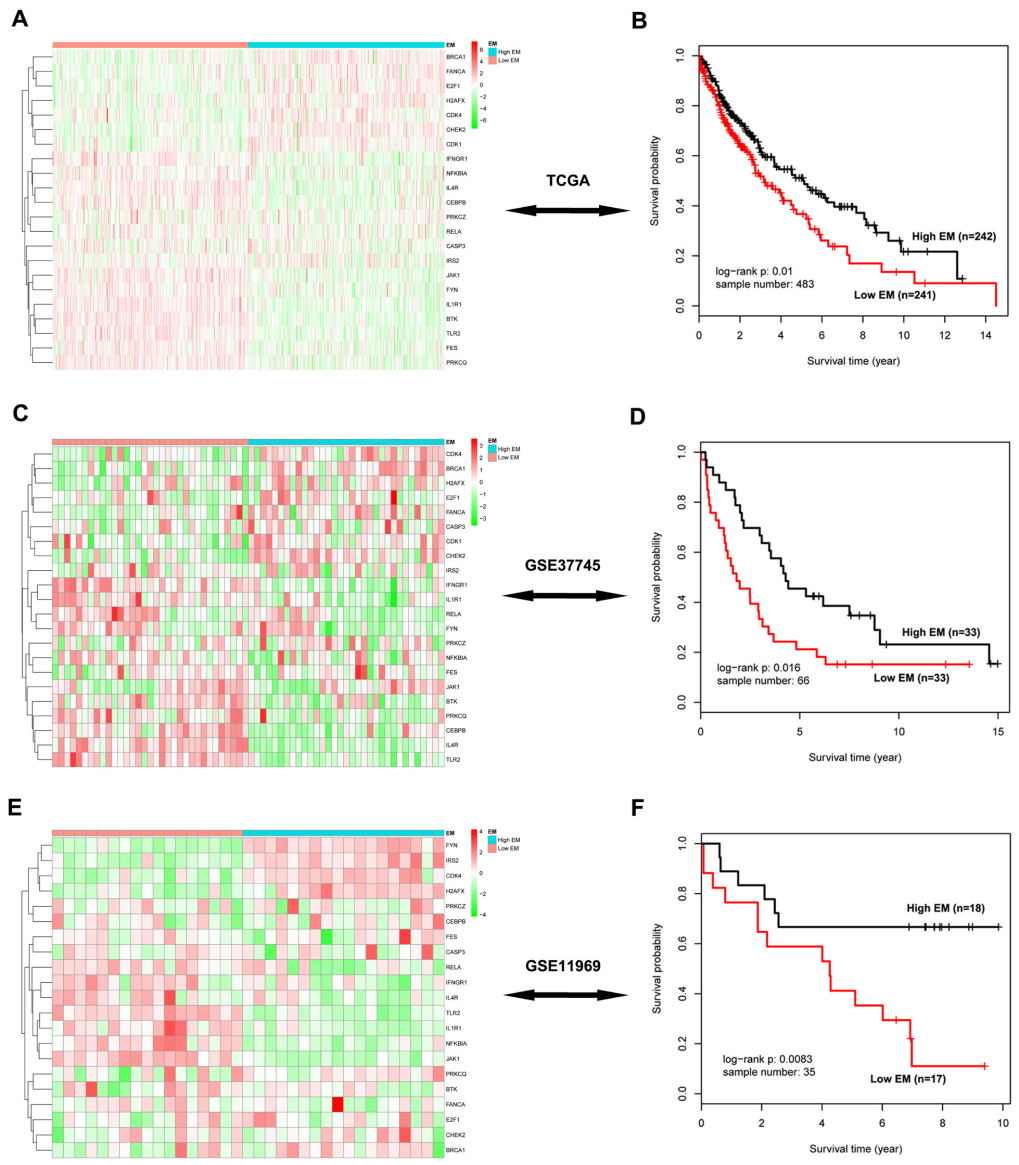
Survival analysis of the significant 22-gene module in three independent testing cohorts **A.** The heatmap of these 22 genes in the TCGA data. Rows represent 22 module genes, which were clustered using an unsupervised clustering algorithm, while columns represent samples, which are divided into two groups according to their corresponding EM value. **B.** Kaplan-Meier survival analysis of these 22 genes in the TCGA data, in which patients are divided into two EM-assigned groups. **C.**-**D.** The heatmap and survival analysis of these module genes in the GSE37745 dataset. **E.**-**F.** The heatmap and survival analysis of these module genes in the GSE11969 dataset.

**Figure 7:**
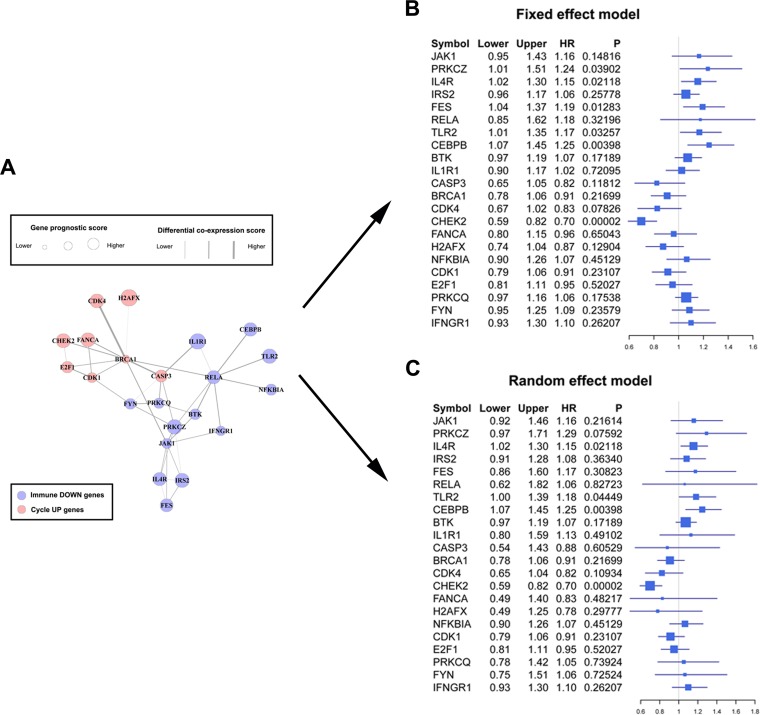
Forest plots of the association between the 22-gene module and overall survival **A.** The significant module composed of 22 genes found using a greedy searching algorithm, including 8 Cycle UP and 14 Immune DOWN genes. Node sized represents gene prognostic score, while edge width represents differential co-expression score. **B.** Forest plot of these 22 module genes with data from the training and three testing cohorts using a fixed effect model. The forest plot shows each gene's official symbol, HR, and *p* value, calculated by pooling all four effect sizes obtained from both the training and testing cohorts. **C.** Forest plot using a random effect model.

We projected the 442 Immune DOWN and Cycle UP genes onto a merged prior knowledge-based biological network; the largest connected component contained 246 genes and 540 interactions (Figure 5). This subnetwork, composed of the consistent DEGs that strongly affected immune response and cell cycle, may contain modules that provide useful prognostic information. To identify these modules (schematic of module identification shown in Supplementary Figure 2), genes and interactions were weighted with prognostic correlation (quantifying the survival correlation) and differentiation of co-expression (quantifying the disruption of gene-to-gene synchronization) during carcinogenesis, respectively. Fifty-three modules were identified using a greedy searching algorithm after merging modules with ≥ 80% overlap. Only 17 modules with significant high scores were found after 10,000 random module sampling (*p* < 0.1). Furthermore, 3 out of these 17 modules (Supplementary Figure 3, Figure 7A) were associated with OS in our training cohort (*p* < 0.1, Table 1). Prognostic evaluation indicated that only the module with *JAK1* as the seed gene (*n* = 22) performed well in three independent testing cohorts (TCGA, *n* = 483, *p* = 0.01, Figure 6A-6B; GSE37745, *n* = 66, *p* = 0.016, Figure 6C-6D; GSE11969, *n* = 35, *p* = 0.0083, Figure 6E-6F). This *JAK1*-centered module, exhibiting consistent prognostic merit in both training and testing cohorts, might be essential in promoting LSQC carcinogenesis. The prognostic performance of the 22 genes of this module in the training cohort is presented in Supplementary Figure 4. The signaling pathway annotation of this 22-gene module (Figure 7A) indicated that these genes were associated with T cell receptor, *p*53 signaling, and apoptosis signaling pathways (Supplementary Table 3, *FDR* < 0.05). Gene and differential co-expression scores for this module are shown in Supplementary Table 4 and Supplementary Table 5, respectively. The expression of *CDK1* is not included in GSE11969, and the survival analysis in this cohort was therefore conducted with the remaining 21 module genes. Meta-analysis was conducted to assess the correlation between individual genes and patients' OS in four datasets (training and testing cohorts) using both a fixed and a random effect model; these two widely used methods pooled the effect sizes of the individual studies into an overall effect size (Figure 7B-7C, the value of *CDK1* in GSE11969 was imputed with the R package “impute”). The Cox proportional hazards regression model was used to evaluate the independence of the prognostic factors in a stepwise manner (Table 2). In each testing cohort, samples for which OS, age, sex and American Joint Committee on Cancer (AJCC) stage information were known were used to perform the analysis. The expression of these 22 module genes was confirmed as an independent prognostic factor in predicting patients' OS (Table 2), suggesting a potential clinical application.

**Table 1:** Associations between clinicopathological characteristics in the training cohort and overall survival

Characteristics	Number	% (range)	*HR* (95%CI)	*p*
Age				
Mean±SD	59.6±9.33	40-77	1.002 (0.962~1.043)	0.933
Sex				
Male	65	94.2	1.150 (0.271~4.882)	0.850
Female	4	5.8	1.000 (reference)	-
T status				
T1+T2	46	66.7	1.000 (reference)	-
T3+T4	23	33.3	1.934 (0.901~4.153)	0.091
N status				
N0	35	50.7	1.000 (reference)	-
N1+N2	34	49.3	1.577 (0.727~3.420)	0.249
AJCC stage				
Stage I	25	36.2	1.000 (reference)	-
Stage II	21	30.5	1.590 (0.568~4.455)	0.377
Stage III	23	33.3	3.032 (1.169~7.866)	**0.023**
Smoking (packs per year)				
Mean±SD	33.5±26.0	0-138	1.007 (0.994~1.020)	0.299

Abbreviations: *SD*, standard deviation; *HR*, hazard ratio; *CI*, confidence interval. Significant *p* values are in bold (*p* < 0.05).

**Table 2:** Univariate and multivariate analyses of overall survival (Cox proportional hazards regression model) in three testing cohorts

Factors	Univariate Cox regression		Multivariate Cox regression	
	*HR* (95% CI)	*p*	*HR* (95% CI)	*p*
***GSE37745***				
Age	1.012 (0.977~1.047)	0.509	-	-
Sex (Male/Female)	0.826 (0.456~1.497)	0.528	-	-
Stage (I+II/III+IV)	2.050 (1.018~4.132)	0.045	2.621 (1.253~5.483)	**0.010**
EM^a^	0.512 (0.293~0.893)	0.018	0.443 (0.247~0.791)	**0.006**
***GSE11969***				
Age	1.030 (0.975~1.088)	0.295	-	**-**
Sex (Male/Female)	1.282 (0.171~9.592)	0.809	-	**-**
Stage (I+II/III+IV)	3.407 (1.395~8.323)	0.007	3.034 (1.233~7.467)	**0.016**
EM^a^	0.289 (0.109~0.765)	0.012	0.321 (0.120~0.863)	**0.024**
***TCGA***				
Age	1.017 (0.999~1.035)	0.057	-	**-**
Sex (Male/Female)	1.109 (0.797~1.542)	0.539	-	**-**
Stage (I+II/III+IV)	1.516 (1.090~2.108)	0.013	1.506 (1.082~2.095)	**0.015**
EM^a^	0.690 (0.518~0.918)	0.011	0.694 (0.522~0.923)	**0.012**

a Samples were divided into two groups based on the eigengene value for the 22-gene module (EM). Significant *p* values are in bold (*p* < 0.05). Abbreviations: *HR*, hazard ratio; *CI*, confidence interval.

## DISCUSSION

MacDonald proposed the concept of biological predeterminism of human cancers, suggesting that clinical outcome can be determined by the intrinsic or destined natural history of cancer [19]. An investigation of a mammary intraepithelial neoplasia outgrowth mouse model indicated that precancerous cells possess malignant potential for latency and metastasis, independent of the accumulation of additional genetic alterations [20]. Additionally, invasive behavior was discovered in precancerous cells, indicating that cancer dissemination may precede tumor formation [21]. These observations suggest that cancer cells may acquire the ability to proliferate and invade largely during precancerous stages. Similarly, our study of colorectal cancer [22] indicated that most differentiated genes in cancer may already be consistently differentiated in precancerous stages.

Consistent DEG analysis indicated that precancerous and cancer samples manifest similar differential gene expression patterns during carcinogenesis (Figure 2G-2J). Thus, DEGs activated or suppressed in both precancerous and cancer stages are likely involved in key regulatory abnormalities that occur during carcinogenesis. GO analysis indicated that consistent DEGs were involved in immune response and cell cycle processes. Tumor-associated cell cycle defects may induce aberrant proliferation as well as genomic and chromosomal instability, which are often mediated by alterations in cyclin-dependent kinase (CDK) activity [23]. Infection and chronic inflammation contribute to an estimated 25% of all cancers worldwide [24]. In developmental biology, the fetus, which in many ways behaves like an allogenic transplant, also evades maternal immune-surveillance through mechanisms similar to those observed in tumors [25]. Indeed, excessive proliferation (activation of cell cycle genes) and immune-surveillance evasion (suppression of immune genes) allow tumors to obtain territorial expansion advantages compared to normal cells.

The association between embryonic development and carcinogenesis [26, 27] makes developmental models useful for studying cancer, in part because they help circumvent potentially misleading complexity caused by tumor heterogeneity [28-30]. Many cellular processes, including epithelial-to-mesenchymal transition (EMT) [31], mesenchymal-to-epithelial transition (MET) [32], and immune-surveillance evasion [25] occur during both embryonic development and carcinogenesis. Currently, there are two theories explaining these similarities. First, cancers may be capable of excessive territorial expansion, migration, and invasion because of genetic or epigenetic changes, both of which also play important roles during normal development [33-35]. Second, tumors may originate from either tissue stem cells or their immediate progeny by diverging from tightly-regulated normal development pathways such that they share characteristics with embryonic cells [36]. The existence of cancer stem cells has been demonstrated, especially in the hematopoietic and colorectal systems [37, 38]. Regardless of the underlying causes, certain pivotal genes are differentially expressed in both embryonic development and carcinogenesis. For example, the activity of *ENAH*, a very important molecule in breast cancer transformation and invasiveness, decreases during mammary gland development, but increases in breast tumors [39]. Additionally, *VICKZ* is thought to be essential for generating and stabilizing the transformed cell phenotype. *VICKZ* expression typically ceases in virtually all tissues soon after birth; however, it is expressed or amplified in at least 12 different kinds of cancer [40]. Therefore, Immune DOWN and Cycle UP genes, which were up-regulated or down-regulated in both cancer and developmental tissues, likely represent core genes that promote the tumor formation.

Notably, Immune DOWN genes were underexpressed in both embryonic development and cancer samples in comparison to normal tissues; Cycle UP genes were overexpressed in both, and in lung adenocarcinoma as well [41]. These findings suggest that cancer spreads by hijacking cellular programs essential for embryonic development. During carcinogenesis, Immune DOWN and Cycle UP genes were drastically differentiated in the precancerous stage. However, after the time point P1, their expression was relatively stable, indicating that most gene expression differentiation occurred in the precancerous stage. Additionally, the Pearson correlation pattern was severely disrupted from development to carcinogenesis (Figure 4C-4F), reflecting the gradual deterioration of gene-to-gene regulatory relationships.

The largest connected component composed of Immune DOWN and Cycle UP genes (Figure 5) showed that these two groups of genes separately formed compact regulatory interactions, and that many regulations existed between the two gene groups. This network described regulatory interactions between two functionally pivotal gene groups that are altered in cancer; dynamic modularity analysis based on this network might help identify core events during carcinogenesis and genes that predict prognosis [42].

Genes in the network were weighted with corresponding prognostic correlation, and interactions were weighted with the differentiation of co-expression, similar to a previous study [43]. In this way, significant modules identified by a greedy searching algorithm [44] contained specific genes that were correlated with OS and affected by co-expression differentiation during carcinogenesis. A module was identified (*n* = 22, Figure 7A) in which expression was significantly correlated with patients' OS in three testing cohorts (Figure 6). This module, containing 8 Cycle UP genes and 14 Immune DOWN genes subject to strong expression alterations and co-expression differentiation, may represent an important mechanism of tumor initiation. The individual prognostic ability of these 22 genes was further illustrated through meta-analysis. Fixed and random-effect models are the most commonly used methods in conducting these meta-analyses, and both use different strategies to pool effect sizes obtained from the individual studies into an overall effect size. The fixed-effect model assumes that differences between the studies are important enough that, during the effect-size pooling process, individual effect sizes should be retained; on the contrary, the random-effect model assumes that individual trial effect sizes are “random” quantities [45, 46].

In summary, expression profiles of human lung embryonic development, precancerous progression, and LSQC samples were analyzed to identify genes with prognostic value. Consistent DEGs differentiated in both precancerous and cancer samples were identified. Up-regulated consistent DEGs were primarily related to cell cycle processes, while down-regulated consistent DEGs were primarily related to immune responses. Furthermore, a significant gene module was identified using a network-based greedy searching algorithm, and the expression of its 22 genes was significantly associated with patients' OS.

## MATERIALS AND METHODS

### Ethics statement

Informed consent was obtained from all donors. This study was conducted in accordance with the ethical standards of the Declaration of Helsinki. The use of human tissue samples and the experimental procedures for this study were reviewed and approved by the Ethics Committee of the Cancer Institute and Hospital, Chinese Academy of Medical Sciences. All experiments were performed in accordance with relevant national and international guidelines.

### Patients and samples

The human developing lung [including whole embryos (WE, *n* = 10) and early (EEL, *n* = 10) and middle embryonic lung (MEL, *n* = 9)] and adult normal lung (NL, *n* = 15) samples were used in our previous investigation (National Center for Biotechnology Information Gene Expression Omnibus (GEO) accession number GSE43767) [41]. Precancerous progression and LSQC cancer samples were collected from 62 patients through bronchoscopy in the Department of Endoscopy, Cancer Hospital, Chinese Academy of Medical Sciences. The biopsy samples included 23 precancerous progression cases [5 cases with mild or moderate dysplasia (P1) and 18 cases with carcinoma in situ (P2)] and 39 lung squamous carcinoma cases (11 Stage I, 13 Stage II, 8 Stage III, and 7 Stage IV cases). Sixty-nine LSQC samples with OS information were obtained *via* surgical excision at the Cancer Institute and Hospital, Chinese Academy of Medical Sciences (Table 1). All tissue samples were snap-frozen in liquid nitrogen immediately after biopsy or surgery and stored at −80°C. A portion of the samples was subjected to pathological analysis performed by two independent and experienced pathologists blind to the experimental purpose. Samples that satisfied the diagnostic criteria for precancerous and neoplastic histology (abnormal cells > 80%) were enrolled. If more than one biopsy sample was taken from the same patient, these samples were pooled.

### RNA isolation and microarray expression profiling

Total RNA was extracted from frozen tissues using TRIzol RNA isolation reagent (Invitrogen, Carlsbad, CA, USA) according to the manufacturer's specifications. RNA integrity was evaluated using a 2100 Bioanalyzer (Agilent Technologies, Santa Clara, USA). If the RNA integrity number was ≥ 6.5, the total RNA was further purified using the RNeasy Mini Kit (Cat No.74106, Qiagen, Germany). RNA concentrations were determined with a NanoDrop ND-1000 Spectrophotometer (NanoDrop Technologies, Wilmington, USA).

After histopathological evaluation and RNA integrity analysis, all samples were purified and analyzed using Agilent microarrays. Total RNA samples from human developmental tissues and cancer samples with OS information were labeled and hybridized to Agilent 4*44K Whole Human Genome Oligo Microarrays (G4112F); precancerous and cancer samples were analyzed using an Agilent SurePrint G3Human GE 8*60K Microarray (G4851B).

### Data preprocessing and normalization

Normalized expression data were extracted with the R package “limma” using the cyclic loess method, and the ComBat algorithm was utilized to eliminate potential batch effects of the 40,894 common probes shared by these two Agilent platforms, as in Clarke et al.'s study [47]. The expression levels of 18,453 genes were defined as the median value of all probes mapping to a particular gene. The raw and processed data are available in the GEO database with the series accession numbers GSE73402 (precancerous and cancer biopsy samples) and GSE73403 (surgically excised cancer samples with OS information).

The mRNA sequencing (RNAseq, *n* = 552) level 3 data for lung squamous carcinoma were retrieved from the Cancer Genome Atlas (TCGA) database (https://tcga-data.nci.nih.gov/tcga/). Fifty-two pairs of RNAseq data were used for GSEA anlaysis. Cancer samples with OS information (*n* = 483) were used for prognostic evaluation. Datasets with OS information (GSE37745 and GSE11969, only LSQC samples were included) were downloaded from the GEO database and used as a testing cohort for prognostic evaluation in conjunction with data from 483 TCGA samples.

### Identification of consistent differentially expressed genes in precancerous progression and cancer samples

Since the majority of differentiated genes in cancer are already consistently differentiated in precancerous stages, consistent DEGs in precancerous and cancer samples were identified to reduce signal noise. An unpaired *t*-test was conducted to identify DEGs between both precancerous progression samples (including P1 and P2, *n* = 23) and normal tissues (*n* = 15), and between cancer samples (*n* = 39) and normal tissues (FDR < 0.01, fold change > 1.5). Genes that were up-regulated or down-regulated in both progression and cancer samples (as compared to normal samples) were regarded as consistent differential DEGs, and were analyzed further.

### Establishing a merged prior knowledge-based biological network

The protein-protein interaction network was downloaded from the Human Protein Reference Database (HPRD), and the Kyoto Encyclopedia of Genes and Genomes (KEGG) network was constructed with the Bioconductor package “KEGGgraph”. The gene regulatory network was established by merging the HPRD and KEGG networks, which included 10,340 nodes and 60,642 edges after self-loops and duplicated edges were eliminated.

### Searching for significant modules

In a given connected biological network, gene *i* was first weighted with *z*i as follows:

$$\text{Zi}=\Phi^{-1}(1-\text{pi})$$ (1)

where *p*i represented the significance of the correlation between the expression value of gene *i* and patients' prognosis, calculated by univariate Cox regression, and Φ^−1^ denoted the inverse standard normal cumulative distribution function (CDF) [48, 49]. Thus, *z*i monotonically increased along with prognostic significance of gene *i*, and followed a standard normal distribution.

Next, the edge between a gene pair (gene *x* and *y*) was weighted to represent the differentiation of co-expression between precancerous progression and cancer stage. Pearson correlation coefficient (*r*) was calculated in each of the two stages and then transformed into a *z*-score value (*z*r) using Fisher's z transformation.

$$\text{Zr}=\frac{1}{2}\text{ln}(\frac{1+r}{1-r})$$ (2)

Then, the differentiation of co-expression (Δ) between the gene pair during carcinogenesis was calculated using the following formula [50]:

$$\Delta k=\frac{\left|\text{ZrPk}-\text{ZrCr}\right|}{\sqrt{\frac{1}{n_{p}-3}+\frac{1}{n_{c}-3}}}$$ (3)

In this formula, *ZrPk* and *ZrCk* represented the transformed Pearson correlation in the progression and cancer stages, respectively; *n*p and *n*c represented the progression and cancer stage sample numbers, respectively. The score *s* of a candidate module, denoted as *g* = (*V*, *E*), was determined using the following formula [51]:

$$S_{g}=\frac{\sum_{v\in V}\text{Zv}+\sum_{k\in E}\Delta k}{\sqrt{m+n}}$$ (4)

where *m* represented the number of nodes (V) and *n* represented the number of edges (E) in module *g*.

Identifying the maximal-scoring connected module can be difficult [49]. In this study, a greedy search was performed to identify modules within the connected biological network for which scores were locally maximal [44, 52]. Candidate modules were seeded with each gene in the connected network and iteratively expanded. In each iteration, the module recruited a neighboring gene within a specified network distance *d* (*d* = 2 in this study, as in Chuang et al.'s study [44]) from the seed. The addition that yielded the maximal score increase was adopted; the search stopped when further additions did not increase the score by more than a specified improvement rate *r* (*r* = 0.1 in this study) [44]. Modules that overlapped by more than 80% in comparison to their sizes were also merged [53, 54].

To determine the statistical significance of a candidate module *M* (including *m* genes), 10,000 random modules with *m* connected genes were sampled, and the 10,000 module scores were used as the null distribution. Modules with *p* value < 0.1 were considered significant [50].

### Survival analysis

We calculated the eigengene of the module (EM) using first principal component across the expression profile of cancer patients. Kaplan-Meier survival analysis and the log-rank test were used to evaluate prognostic differences between the two EM-assigned groups [15, 55-57]. The Cox proportional hazards regression model was used to evaluate the independence of the prognostic factors in a stepwise manner. Samples in each dataset with complete patient age, sex, stage, and OS information were used for Cox analysis, and a value of *p* < 0.05 was regarded as significant.

## SUPPLEMENTARY MATERIAL FIGURES AND TABLES






