# Supplementary material for: A network-based method for identifying prognostic gene modules in lung squamous carcinoma
Source: Oncotarget. 2016 Feb 23;7(14):18006–20. doi: 10.18632/oncotarget.7632 (PMC4951267; doi:10.18632/oncotarget.7632)
Supplement: Supplementary file 3 [file oncotarget-07-18006-s003.pdf]

A network-based method for identifying prognostic gene modules in lung squamous carcinoma

Supplementary Material

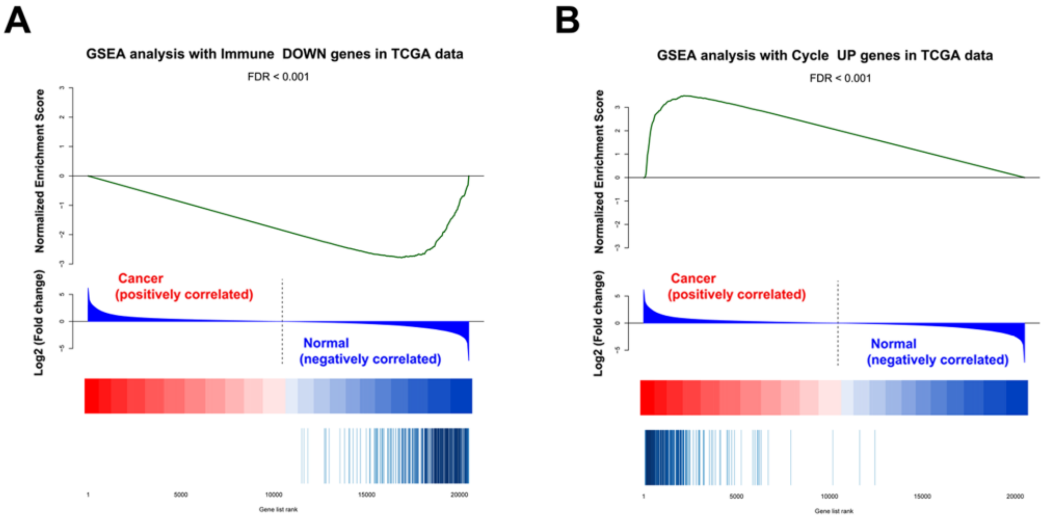

Supplementary Figure 1: GSEA analyses with Immune DOWN and Cycle UP genes in TCGA data. (A) Immune DOWN genes were significantly down-regulated in cancer samples in comparison to adjacent normal tissue. (B) Cycle UP genes were significantly up-regulated in cancer samples in comparison to adjacent normal tissue, indicating that these consistent DEGs had consistent differential expression pattern in cancer and adjacent normal tissue.

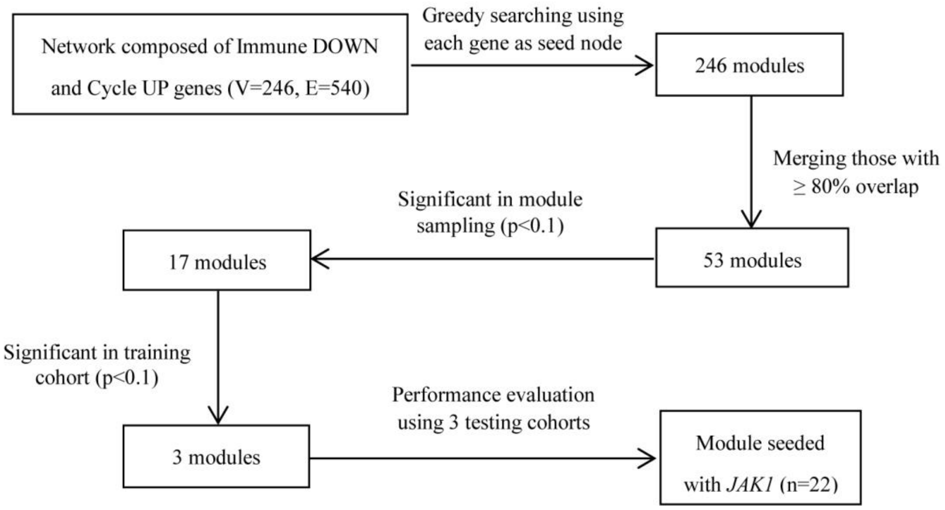

Supplementary Figure 2: Schematic of module selection procedures.

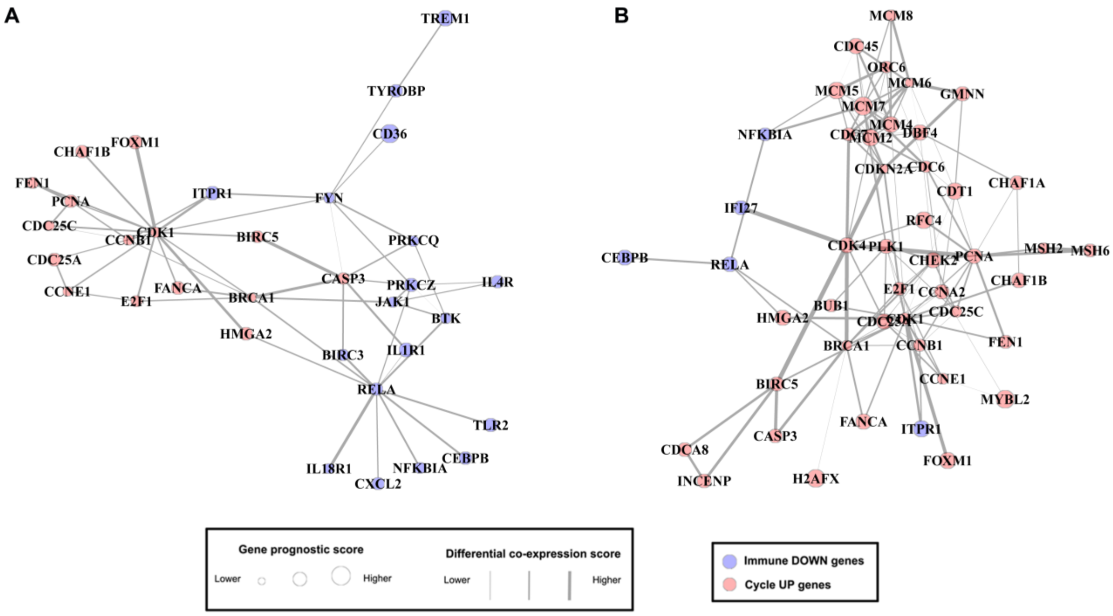

Supplementary Figure 3: The remaining two significant modules didn’t perform well in testing cohorts.

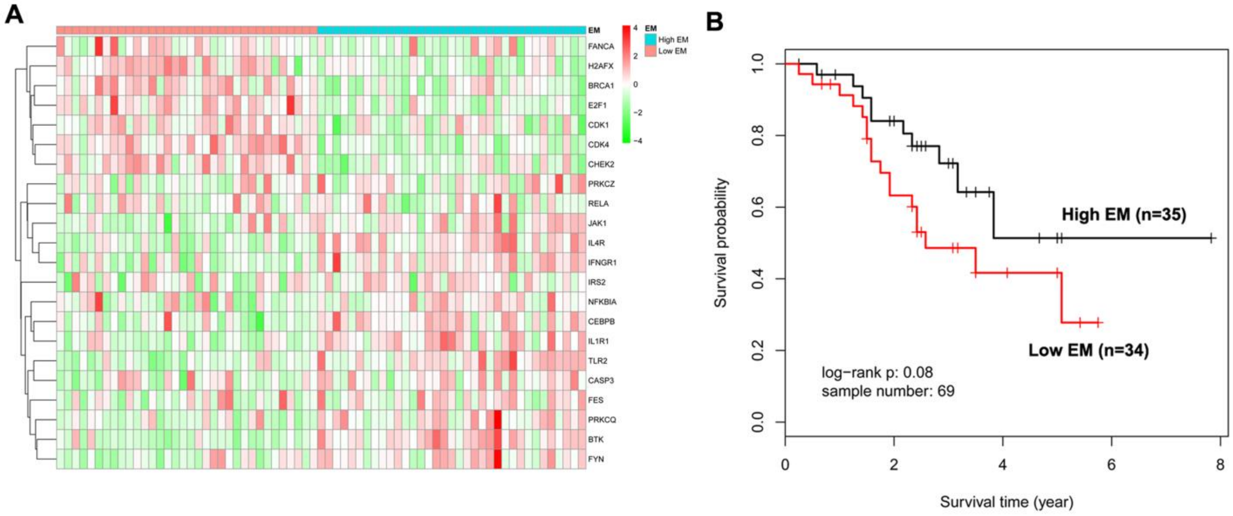

Supplementary Figure 4: Survival analysis of the significant 22-gene module in training cohort. **A.** The heatmap of these 22 genes in training data. Rows represent 22 module genes, while columns represent samples, which are divided into two groups according to their corresponding EM value. **B.** Kaplan–Meier survival analysis of these 22 genes in training data, of which the patients are divided into two EM-assigned groups.

Supplementary Table 1: GO analysis of 1025 consistent downregulated DEGs using DAVID

Supplementary Table 2: GO analysis of 1376 consistent downregulated DEGs using DAVID

Supplementary Table 3: KEGG pathways associated with these 22 genes by gene annotation enrichment analysis using DAVID

Term	Count	%	Pvalue	Enrichment	FDR
hsa04660:T cell receptor signaling pathway	5	22.727	0.001	11.771	0.006
hsa04920:Adipocytokine signaling pathway	4	18.182	0.002	15.179	0.018
hsa04115:p53 signaling pathway	4	18.182	0.002	14.956	0.018
hsa05212:Pancreatic cancer	4	18.182	0.002	14.125	0.022
hsa05220:Chronic myeloid leukemia	4	18.182	0.003	13.560	0.024
hsa05222:Small cell lung cancer	4	18.182	0.003	12.107	0.033
hsa04210:Apoptosis	4	18.182	0.004	11.690	0.037
hsa05200:Pathways in cancer	6	27.273	0.006	4.651	0.056
hsa04110:Cell cycle	4	18.182	0.011	8.136	0.098
hsa05120:Epithelial cell signaling in Helicobacter pylori infection	3	13.636	0.026	11.217	0.226
hsa04662:B cell receptor signaling pathway	3	13.636	0.031	10.170	0.265
hsa05215:Prostate cancer	3	13.636	0.043	8.570	0.346
hsa04620:Toll-like receptor signaling pathway	3	13.636	0.054	7.552	0.415
hsa04722:Neurotrophin signaling pathway	3	13.636	0.077	6.151	0.541
hsa04650:Natural killer cell mediated cytotoxicity	3	13.636	0.087	5.735	0.587
hsa04530:Tight junction	3	13.636	0.088	5.692	0.592
hsa04630:Jak-STAT signaling pathway	3	13.636	0.113	4.921	0.687
hsa05219:Bladder cancer	2	9.091	0.146	12.107	0.784
hsa04062:Chemokine signaling pathway	3	13.636	0.153	4.079	0.801
hsa04930:Type II diabetes mellitus	2	9.091	0.162	10.819	0.820
hsa05223:Non-small cell lung cancer	2	9.091	0.184	9.417	0.861
hsa04623:Cytosolic DNA-sensing pathway	2	9.091	0.187	9.245	0.866
hsa04621:NOD-like receptor signaling pathway	2	9.091	0.208	8.202	0.897
hsa05214:Glioma	2	9.091	0.211	8.071	0.900
hsa04622:RIG-I-like receptor signaling pathway	2	9.091	0.235	7.162	0.926
hsa05218:Melanoma	2	9.091	0.235	7.162	0.926
hsa05416:Viral myocarditis	2	9.091	0.235	7.162	0.926
hsa04664:Fc epsilon RI signaling pathway	2	9.091	0.255	6.519	0.943
hsa04060:Cytokine-cytokine receptor interaction	3	13.636	0.256	2.911	0.944
hsa04010:MAPK signaling pathway	3	13.636	0.263	2.857	0.949
hsa04640:Hematopoietic cell lineage	2	9.091	0.277	5.913	0.957
hsa04360:Axon guidance	2	9.091	0.387	3.942	0.991
hsa04910:Insulin signaling pathway	2	9.091	0.401	3.767	0.993

Supplementary Table 4: Gene scores of the 22 genes in the significant module with JAK1 as the seed.

Gene symbol	Entrez identifier	Gene score
JAK1	3716	-0.310
PRKCZ	5590	1.518
IL4R	3566	0.967
IRS2	8660	1.347
FES	2242	0.889
RELA	5970	0.963
TLR2	7097	1.455
CEBPB	1051	1.092
BTK	695	-0.028
IL1R1	3554	1.903
CASP3	836	0.460
BRCA1	672	-1.080
CDK4	1019	1.220
CHEK2	11200	1.287
FANCA	2175	1.250
H2AFX	3014	2.296
NFKBIA	4792	0.146
CDK1	983	-0.357
E2F1	1869	0.253
PRKCQ	5588	-0.203
FYN	2534	-0.053
IFNGR1	3459	-0.531

Supplementary Table 5: Interaction score of all the edges in the significant 22-gene module with JAK1 as the seed.

Interactor1	Interactor2	Interaction score
JAK1	PRKCZ	1.672
JAK1	IL4R	1.216
JAK1	IRS2	1.127
JAK1	FES	1.107
JAK1	BTK	1.645
JAK1	BRCA1	1.946
JAK1	IFNGR1	1.417
PRKCZ	IL4R	0.901
PRKCZ	IRS2	0.427
PRKCZ	RELA	1.264
PRKCZ	BTK	0.12
PRKCZ	CASP3	1.378
PRKCZ	FYN	1.451
IL4R	IRS2	0.336
IL4R	FES	0.354
IRS2	FES	1.224
RELA	TLR2	2.119
RELA	CEBPB	2.183
RELA	BTK	2.061
RELA	IL1R1	0.504
RELA	BRCA1	1.788
RELA	NFKBIA	2.033
RELA	IFNGR1	1.803
BTK	PRKCQ	1.180
IL1R1	CASP3	2.352
CASP3	BRCA1	2.973
CASP3	PRKCQ	1.725
CASP3	FYN	0.236

**BRCA1**	**CDK4**	**4.272**
**BRCA1**	**CHEK2**	**2.005**
**BRCA1**	**FANCA**	**1.966**
**BRCA1**	**H2AFX**	**0.250**
**BRCA1**	**CDK1**	**1.423**
**BRCA1**	**E2F1**	**1.535**
**CHEK2**	**E2F1**	**0.602**
**FANCA**	**CDK1**	**1.648**
**CDK1**	**E2F1**	**2.058**
**CDK1**	**FYN**	**1.340**
**PRKCQ**	**FYN**	**1.815**
